# CHALLENGES FOR IMPLEMENTING PRAGMATIC COMMUNICATION INTERVENTIONS IN NURSING HOME SETTINGS

**DOI:** 10.1093/geroni/igz038.1327

**Published:** 2019-11-08

**Authors:** Kristine N Williams, Clarissa Shaw, Carissa K Coleman

**Affiliations:** 1 University of Kansas Medical Center, Kansas City, Kansas, United States; 2 University of Iowa College of Nursing, Iowa City, Iowa, United States; 3 University of Kansas School of Nursing, Kansas City, Kansas, United States

## Abstract

Interventions to improve nursing home care have been developed and tested. However readily disseminated interventions are lacking. Barriers include low staffing levels contributing to limited time for education and high turnover of direct care and administrative staff. Educational interventions must be accessible to accommodate busy staff. Meaningful outcome measures are needed and interventions must fit varied nursing home sizes, ownership, resident population, and regions. Changing Talk Online (CHATO) was adapted from the effective, yet time-intensive, Changing Talk program addressing nursing home staff communication. The original classroom-based program significantly improved staff communication with residents and resulted in subsequent reductions in resident behavioral and psychological symptoms of dementia. Strategies for marketing and recruiting nursing homes and to engage administrators and staff will be discussed as implemented in the Changing Talk Online (CHATO) R61 trial. Approaches addressing unique nursing home challenges to implementation are essential for successful dissemination to improve care.

